# Peanut skin extract ameliorates the symptoms of type 2 diabetes mellitus in mice by alleviating inflammation and maintaining gut microbiota homeostasis

**DOI:** 10.18632/aging.103521

**Published:** 2020-07-22

**Authors:** Lan Xiang, Qiaobei Wu, Hiroyuki Osada, Minoru Yoshida, Wensheng Pan, Jianhua Qi

**Affiliations:** 1 College of Pharmaceutical Sciences, Zhejiang University, Hangzhou, P. R. China; 2 Chemical Biology Research Group, RIKEN Center for Sustainable Resource Science, Wako-Shi, Saitama, Japan; 3 Chemical Genomics Research Group, RIKEN Center for Sustainable Resource Science, Wako, Saitama, Japan; 4 Department of Biotechnology and Collaborative Research Institute for Innovative Microbiology, The University of Tokyo, Bunkyo-ku, Tokyo, Japan; 5 Department of Gastroenterology, Zhejiang Provincial People's Hospital, People's Hospital of Hangzhou Medical College, Hangzhou, Zhejiang Province, China

**Keywords:** peanut skin extract (PSE), gut microbiota, type 2 diabetes, anti-inflammation, mice

## Abstract

In this study, mice with type 2 diabetes mellitus (T2DM) induced by high-fat diet were used to investigate the antidiabetic effect and mechanism of action of peanut skin extract (PSE). Results revealed that the fasting blood glucose, body weight, and food intake of mice with T2DM significantly decreased after they were given PSE. The effects of 80 mg/kg PSE were similar to those of 140 mg/kg metformin (MET). The glucose tolerance and insulin sensitivity of the mice also improved. The composition of intestinal microflora in the mice significantly changed after PSE administration. In particular, no *Actinobacteria* was detected in the PSE-treated group, and the ratio of *Firmicutes* to *Bacteroidetes* was remarkably reduced. PSE also increased the abundance of gut microbiota involved in fatty acid biosynthesis, lipid biosynthesis, and sucrose metabolism. The abundance of gut microbiota related to aminoacyl-tRNA biosynthesis also decreased. Lipopolysaccharide, interleukin (IL)-6, IL-1β and tumor necrosis factor-α in the blood, liver and adipose tissue were reduced by PSE. Similarly, the mRNA expression levels of IkappaB kinase and nuclear factor kappaB in the hypothalamus were reduced by PSE. These results suggested that PSE and MET elicited significant antidiabetic effects by maintaining gut microbiota and inhibiting inflammation.

## INTRODUCTION

With the development of scientific techniques, new insights into intestinal microorganisms have been provided. For example, they participate in food digestion and link the external environment to their hosts. They can accurately sense changes in external signals and food components. They can also produce metabolic small molecules that affect the metabolic homeostasis of adipose tissues and organs, such as the liver, brain, and heart. As such, the disorder of intestinal microorganisms is closely related to many diseases, such as obesity, diabetes, cancer, cardiovascular diseases, digestive tract, and neuronal degeneration diseases [1–5].

Gut microbial dysbiosis associated with type 2 diabetes mellitus (T2DM) and obesity has been widely explored [1–2]. The compositions and proportion of gut microbiota in patients with these diseases are obviously altered compared with those of healthy people. For instance, the abundance of *Firmicutes* and *Actinobacteria* significantly increases, whereas the abundance of *Bacteroidetes* decreases [6]. Furthermore, diabetes induced by high-fat diet (HFD) reduces intestinal integrity and increases endotoxemia due to the translocation of bacterial lipopolysaccharide (LPS) from the gut microbiota to the blood [7]. LPS also induces systemic low-grade inflammation and insulin resistance of the central nervous system and peripheral tissues, such as the liver and adipose tissues [8].

Pro-inflammatory cytokines, including interleukin (IL)-1, IL-6, and tumor necrosis factor (TNF)-α, are related to insulin resistance and metabolic syndromes, such as obesity and T2DM [9]. IL-6 has been identified as a pathogenic marker of hepatic diseases. It activates proteins known as suppressors of cytokine signaling 3 (SOCS3) via the phosphorylation of Janus kinase 2 and the signal transducer and activator of transcription 3 [1, 10]. SOCS3 induced by IL-6 inhibits hepatic insulin signaling by attenuating the activation and degradation of insulin receptor substrate-1,2 (IRS-1,2) [11–13].

In China, peanut skin as a Chinese herbal medicine is used to treat hemophilia, thrombocytopenic purpura, and hepatic hemorrhage. Peanut skin extract (PSE) is rich in phenolic compounds and polysaccharides [14, 15]. Phenolic compounds are effective against diabetes, obesity, cancer, cardiovascular diseases, and inflammation [16–21]. In our previous studies, a mixture of PSE and fish oil can improve the memory and learning ability of normal mice [22]. PSE also elicits an anti-obesity effect on mice [15]. Polysaccharides from natural sources have good antidiabetic effects [23, 24]. As such, we speculated that PSE exhibited an antidiabetic effect on T2DM. To verify this speculation, we constructed a T2DM mouse model with HFD and used it to investigate the pharmacodynamics and mechanism of action of PSE. Herein, we reported that PSE elicited antidiabetic effects similar to those of metformin (MET) by inhibiting inflammation and maintaining the homeostasis of intestinal microflora.

## RESULTS

### Alleviation of the clinical symptoms and energy metabolism disorder of mice with T2DM via oral PSE administration

**Figure 1:**
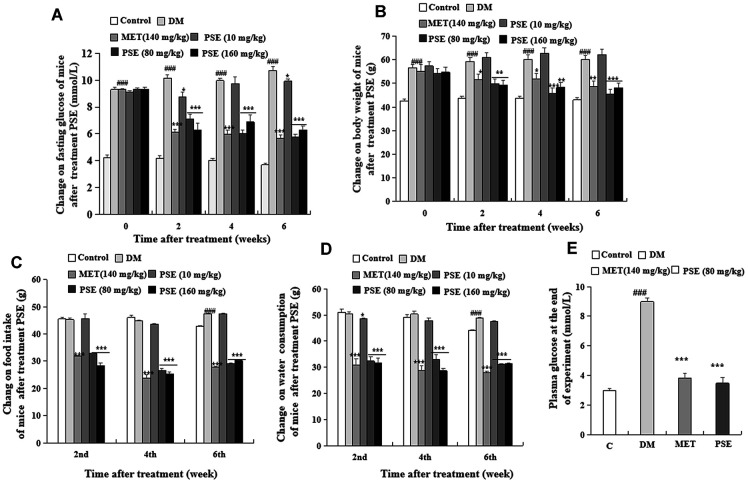
**Anti-diabetic effects of PSE on HFD-induced diabetic mice.** The changes of fasting blood glucose (**A**), body weight (**B**), food intake (**C**), water consumption (**D**), and plasma glucose at the end of experiment (**E**) of diabetic mice after administering PSE at 10, 80, and 160 mg/kg for 6 weeks. Each value was expressed as the mean ± SEM of 10 mice. ^#^, ^##^, and ^###^ represent significant difference compared with the control group at p < 0.05, p < 0.01, and p < 0.001, respectively. ^*^, ^**^, and ^***^ indicate significant difference compared with the DM group at p < 0.05, p < 0.01, and p < 0.001, respectively.

The change in fasting blood glucose, body weight, food intake, and water consumption of mice with T2DM after they were orally administered with PSE are shown in Figures 1A–1D. The fasting blood glucose (measured with a test paper; Figure 1A) and body weight (Figure 1B) of the mice in the diabetic model (DM) group were significantly higher than those of the normal mice in all the experimental periods (p < 0.001 and p < 0.001). However, the food intake (Figure 1C) and water consumption (Figure 1D) of the mice with T2DM increased on the 6^th^ week (p < 0.001 and p < 0.001). Meanwhile, these parameters in the mice with T2DM significantly decreased after 6 weeks of administration of 80 and 160 mg/kg PSE and 140 mg/kg MET (Figures 1A–1D; p < 0.001). At the end of the experiment, fasting blood glucose was measured again with the biochemical analyzer, and the results revealed that this parameter obviously decreased. The effect of 80 mg/kg PSE was similar to that of 140 mg/kg MET (Figure 1E; p < 0.001). These results suggested that PSE improved the clinical symptoms of mice with T2DM and elicited the same effects as those of MET.

The weights of the liver, epididymal fat, heart, spleen, pancreas, and kidney of the mice with T2DM after PSE treatment are shown in Supplementary Figure 1. HFD significantly increased the weights of the liver, epididymal fat, heart, pancreas, and kidney of the mice with T2DM (p < 0.001, p < 0.01, p < 0.01, p < 0.01, and p < 0.01, respectively). Furthermore, 80 and 160 mg/kg PSE inhibited obesity in these organs, and their weights returned to normal levels (Supplementary Figure 1; p < 0.001, p < 0.01, p < 0.05, p < 0.001, and p < 0.001). However, the weights of these organs and the adipose tissue of the mice with T2DM were not affected by 10 mg/kg PSE. The blood biochemical markers were also examined. The plasma TG and TC of the mice with T2DM significantly increased compared with those of the normal control group (Supplementary Table 2; p < 0.01 and p < 0.05). The plasma TG and TC of the groups treated with 80 mg/kg PSE and 140 mg/kg MET significantly decreased (Supplementary Table 2; p < 0.01 and p < 0.05). The plasma AST and ALT showed no significant change compared with those of the normal and pathological control groups (Supplementary Table 2). These results indicated that PSE treatment alleviated the symptoms such as hyperphagia, polydipsia, energy metabolism disorder, and hyperglycemia in the mice with HFD-induced T2DM. PSE in the dose range that caused a significant effect did not trigger a toxic response from the mice. The effect of 80 mg/kg PSE was similar to that of 160 mg/kg PSE, so 80 mg/kg PSE was selected to analyze the mechanism and fecal microflora via 16S RNA sequencing.

### PSE-induced improvement of the glucose and insulin tolerance of mice with T2DM

**Figure 2:**
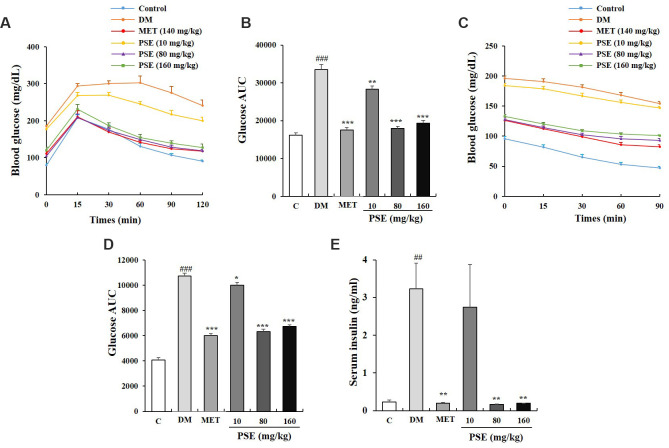
**Effects of PSE on tolerance of glucose and insulin in HFD-induced diabetic mice.** The change on fasting blood glucose (**A**) and AUC (**B**) of HFD-induced diabetic mice during OGTT after administering PSE at 10, 80, and 160 mg/kg for 6 weeks. The change on fasting blood glucose (**C**) and AUC (**D**) of HFD-induced diabetic mice during ITT after administering PSE at 10, 80, and 160 mg/kg for 6 weeks. The change on serum insulin (**E**) of HFD-induced diabetic mice after administering PSE at 10, 80, and 160 mg/kg for 6 weeks. Each value was expressed as the mean ± SEM of 10 mice. ^##^ and ^###^ represent significant difference compared to the control group at p < 0.01 and p < 0.001, respectively. ^*^, ^**^, and ^***^ indicate significant difference compared to the DM group at p < 0.05, p < 0.01 and p < 0.001, respectively.

Hyperglycemia, hyperinsulinemia, and glucose tolerance are associated with obesity and metabolic syndrome [25]. After the oral administration of PSE for 5 weeks, an oral glucose tolerance test (OGTT) and an insulin tolerance test (ITT) were performed to detect the glucose and insulin tolerance of the mice with T2DM. The OGTT results are shown in Figures 2A and 2B. The blood glucose levels of the groups with diabetes at time points 0, 15, 60, 90, and 120 min and the area under the curve (AUC) were higher than those of the normal control group (Figures 2A and 2B; p < 0.001). These indices were significantly decreased by the PSE at all the tested doses and MET at the indicated dose (Figures 2A and 2B; p < 0.01 and p < 0.001). The ITT experiment also revealed that the blood glucose, AUC, and insulin of the DM group were higher than those of the normal control group (Figures 2C–2E; p < 0.001). However, these characteristics of the mice with T2DM were obviously improved by PSE and MET (Figures 2C–2E; p < 0.05 and p < 0.01). These results suggested that PSE treatment alleviated the clinical symptoms of mice with T2DM by increasing glucose tolerance and enhancing insulin sensitivity.

### Sequences and operational taxonomic units of the stool samples of normal, diabetic, PSE-treated, and MET-treated mice

The disorder of gut microflora composition is associated with obesity and diabetes [1, 7]. In our study, Illumina high-throughput sequencing was conducted to read the 16S RNA sequences of the V4 region of 35 fecal microflora samples of the control, DM, 140 mg/kg MET treatment, and 10 and 80 mg/kg PSE treatment groups and to understand whether PSE affected the gut microbiota of mice. Each group had seven samples. Operational taxonomic units (OTUs) were classified on the basis of 97% similarity, and OTU1 with few sequences was removed (i.e., the number of sequences in OTUs was less than 2). The tag numbers of the control, DM, MET-treated, and PSE-treated groups were 72,644 ± 466.3, 74,290 ± 603.1, 71,807 ± 563.8, 72,780 ± 583.9, and 71,990 ± 458.3, respectively (Supplementary Table 3). The averages of the number of OTUs in each group were 512 ± 19.4, 511 ± 16.7, 524 ± 13.8, 521 ± 36.6, and 438 ± 32.3, respectively (Supplementary Table 3). The number of tags and OTUs of each group did not have significant changes.

**Figure 3:**
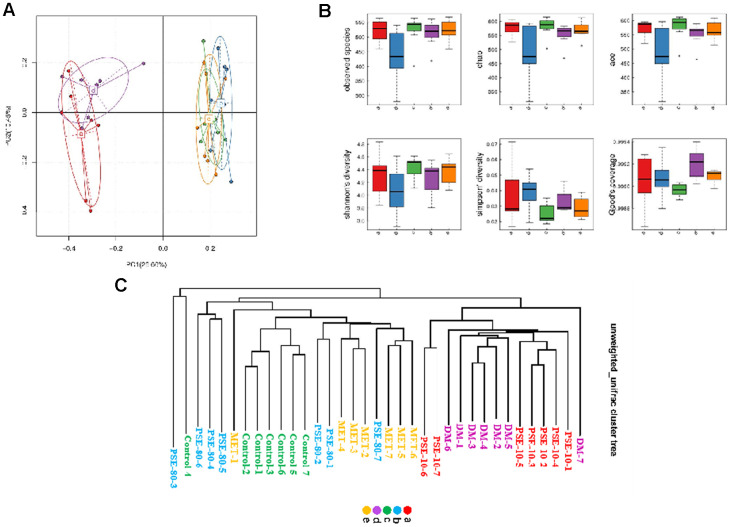
**Results of PCA analysis based on OTU abundance, inter-group alpha diversity box map and multivariate analysis of variance of PCoA.** The results of PCoA analysis based on OTU abundance (**A**) and inter-group alpha diversity box map (**B**). Multivariate analysis of variance of PCoA matrix scores (**C**). Letters a, b, c, d, and e in graph represent 10 mg/kg PSE-treated, 80 mg/kg PSE-treated, normal control, diabetes model, and 140 mg/kg MET-treated groups, respectively. The dots in the figure represent each sample. Different colours represent different groups of samples.

The results of the PCA analysis based on OTU abundance and intergroup alpha diversity box map are given in Figures 3A and 3B. Letters a, b, c, d, and e in the graph represent the 10 mg/kg PSE-treated, 80 mg/kg PSE-treated, normal control, DM, and 140 mg/kg MET-treated groups, respectively. The results showed that the normal control and DM groups could be distinguished clearly on the basis of the gut microbiota composition. Similar to MET, 80 mg/kg PSE significantly affected the gut microbiota composition of the mice with T2DM (Figure 3A). The results of the multivariate ANOVA of the PCoA matrix scores indicated a statistically significant separation between the microbiota of the control group and the DM group. Significant separations were also observed in the DM, MET, and 80 mg/kg PSE groups (Figure 3C). However, the gut microbiota of the mice in the 10 mg/kg PSE-treated group did not significantly differ from that of the mice with diabetes (Figure 3C). This outcome was consistent with the weak effects observed in the mice treated with 10 mg/kg PSE (Figures 1 and 2). These results suggested that PSE significantly regulated the intestinal microflora.

### Regulatory effects of PSE on gut microbiota

**Figure 4:**
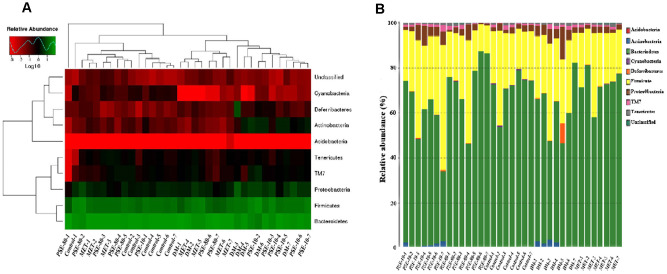
**Effect of PSE on gut-microbiota composition of type 2 diabetic mice-induced by high-fat diet at *phyma* level.** The heatmap of class level species abundance (**A**). Histogram of species profiling at the *phyma* classification level (**B**). Column height represents relative abundance.

Species and abundance analysis was conducted at phylum, class, order, family, genus, and species levels to further understand how PSE regulated gut microbiota. For 35 samples, 9 phyla, 13 classes, 14 orders, 20 families, 22 genera, and 10 species were obtained. Nine phyla, namely, *Acidobacteria*, *Actinobacteria*, *Bacteroidetes*, *Cyanobacteria*, *Deferribacteres*, *Firmicutes*, *Proteobacteria*, *TM7*, and *Tenericutes*, were examined (Figures 4A and 4B). The abundance of *Actinobacteria*, *Proteobacteria*, and *Deferribacteres* of the DM group significantly increased compared with that of the control group (p < 0.001, p < 0.05, and p < 0.001, respectively). The abundance of *Bacteroidetes* and *Cyanobacteria* of the DM group clearly decreased compared with that of the control group (p < 0.05 and p < 0.001, respectively). After 80 mg/kg PSE and 140 mg/kg MET were orally administered, *Actinobacteria* was not detected in the PSE- and MET-treated groups (Figure 4B). Moreover, the ratio of *Firmicutes* to *Bacteroidetes* of the DM group significantly increased. Although no effect was observed in 10 mg/kg PSE-treated group, 80 mg/kg PSE and 140 mg/kg MET significantly decreased the ratio of *Firmicutes* to *Bacteroidetes* and allowed the gut microbiota of the mice with T2DM to recover to normal level.

Thirteen classes, namely, *4C0d-2*, *Actinobacteria*, *Bacilli*, *Bacteroidia*, *Betaproteobacteria*, *Clostridia*, *Deferribacteres*, *Deltaproteobacteria*, *Epsilonproteo-bacteria*, *Erysipelotrichi*, *Gammaproteobacteria*, *Mollicutes*, and *TM7-3*, were investigated (Supplementary Figures 2A and 2B). The abundance of *Actinobacteria*, *Betaproteobacteria*, *Deltaproteo-bacteria*, *Erysipelotrichi*, and *Deferribacteres* of the DM group significantly increased compared with that of the control group (p < 0.0001, p < 0.01, p < 0.01, p < 0.01, and p < 0.01, respectively). By contrast, the abundance of *Bacilli*, *Bacteroidia*, *Chloroplast*, *Gammaproteobacteria*, and *4C0d-2* decreased (p < 0.001, p < 0.05, p < 0.001, p < 0.01, and p < 0.01, respectively). These effects were reversed by treating the mice with 80 mg/kg PSE and 140 mg/kg MET. However, 10 mg/kg PSE-treated group did not have this effect.

Fourteen orders, namely, *Bacteroidales*, *Bifidobacteriales*, *Burkholderiales*, *CW040*, *Campylobacterales*, *Clostridiales*, *Deferribacterales*, *Desulfovibrionales*, *Enterobacrerides*, *Erysipelotrichales*, *Lactobacillales*, *RF39*, *Turicibacterales*, and *YS2*, were analyzed (Supplementary Figures 3A and 3B). The abundance of *Bacillales*, *Turicibacterales*, *Burkholderiales*, *Bifidobacteriales*, *Deferribacterales*, *Desulfovibrionales*, and *Erysipelotrichales* of the DM group significantly increased compared with that of the control group (p < 0.05, p < 0.0001, p < 0.0001, p < 0.0001, p < 0.0001, p < 0.0001, and p < 0.0001, respectively). By contrast, the abundance of *Bacteroidales*, *Enterobacteriales*, *Streptophyta*, *YS2*, and *Lactobacillales* of the DM group significantly decreased compared with that of the control group (p < 0.05, p < 0.01, p < 0.01, and p < 0.0001, respectively). The normal level of the gut microflora of the mice with T2DM was recovered after they were treated with 80 mg/kg PSE and 140 mg/kg MET. However, the abundance of *Lactobacillales* significantly increased in the 10 mg/kg PSE-treated group.

Twenty families, namely, *Alcaligenaceae*, *Bacteroidaceae*, *Bifidobacteriaceae*, *Deferribacteraceae*, *Desulfovibrionaceae*, *Enterobacteriaceae*, *Erysipelotri-chaceae*, *F16*, *Helicobacteraceae*, *Lachnospiraceae*, *Lactobacillaceae*, *Mogibacteriaceae*, *Odoribacteraceae*, *S24-7*, *Turicibacteraceae*, *Paraprevolellaceae*, *Porphy-romobbnadaceae*, *Prevoellaceae*, *Rikenellaceae*, and *Ruminococcaceae*, were analyzed (Supplementary Figures 4A and 4B). The abundance of *Alcaligenaceae*, *Deferribacteraceae*, *Bifidobacteriaceae*, *Desulfovibrio-naceae*, *Erysipelotrichaceae*, *Eubacteriaceae*, *Mogi-bacteriaceae*, *Peptococcaceae*, and *Peptostrep-tococcaceae* of the DM group significantly increased (p < 0.0001, p < 0.0001, p < 0.001, p < 0.0001, p < 0.001, p < 0.001, p < 0.001, p < 0.001, and p < 0.001, respectively). By contrast, the abundance of Clostridiaceae, Enterobacteriaceae, Lactobacillaceae, Rikenellaceae, Odoribacteraceae, and Prevotellaceae of the DM group significantly decreased (p < 0.0001, p < 0.0001, p < 0.001, p < 0.0001, p < 0.0001, and p < 0.001). These changes in the abundance of the microflora of the mice in the DM group were reversed by the treatment with 80 mg/kg PSE and 140 mg/kg MET. Furthermore, *Turicibacteraceae* was found in the DM group. However, this effect was not observed in the 10 mg/kg PSE-treated group.

Twenty-two genera, namely, *AF12*, *Allobaculum*, *Bacteroides*, *Bifidobacterium*, *Clostridium*, *Coprococcus*, *Desulfovibrio*, *Escherichia*, *Flexispira*, *Helicobacter*, *Lactobacillus*, *Mucispirillum*, *Odoribacter*, *Oscillospira*, *Parabacteroides*, *Paraprevotella*, *Prevotella*, *Rikenella*, *Roseburia*, *Ruminococcus*, *Sutterella*, and *Turicibacter*, were checked (Supplementary Figures 5A and 5B). The abundance of *AF12*, *Allobaculum*, *Bifidobacterium*, *Christensenella*, *Coprobacillus*, *Flexispira*, *Mucispirillum*, *Odoribacter*, *Turicibacter*, *Anaerofustis*, and *Sutterella* of the DM group significantly increased compared with that of the control group (p < 0.05, p < 0.0001, p < 0.001, p < 0.0001, p < 0.0001, p < 0.01, p < 0.001, p < 0.0001, p < 0.0001, p < 0.0001, and p < 0.0001, respectively). The abundance of *Escherichia*, *Lactobacillus*, *Lactococcus*, *Odoribacter*, *Paraprevotella*, *Prevotella*, and *Rikenella* of the DM group was lower than that of the control group (p < 0.05, p < 0.001, p < 0.05, p < 0.0001, p < 0.0001, p < 0.05, and p < 0.01, respectively). These changes in abundance of gut microbiota at genera were amended after the mice were treated with 80 mg/kg PSE and 140 mg/kg MET. However, 10 mg/kg PSE did not have the same effect.

Ten species, namely, *Bacteroides acidifaciens*, *Bifidobacterium pseudolc*, *Clostridium methylpentos*, *Desulfovibrio C21 c2*, *Escherichia coli*, *Lactobacillus reuteri*, *Mucispirillum schaedleri*, *Parabacteroides distasor*, *Ruminococcus flavefacie*, and *Ruminococcus gnavus*, were investigated (Supplementary Figures 6A and 6B). The abundance of *B. acidifaciens*, *Conterminous flavefaciens*, *Ruminococcus gnavus*, *Lactobacillus reuteri*, and *Lactococcus garvieae* of the DM group decreased (p < 0.0001, p < 0.05, p < 0.01, p < 0.01, and p < 0.05, respectively), whereas the abundance of *Bifidobacterium pseudolc*, *Clostridium colinum*, *Clostridium hungatei*, *Mucispirillum schaedleri* (p < 0.001, p < 0.01, p < 0.05, and p < 0.001, respectively) of the DM group increased. After 80 mg/kg PSE and 140 mg/kg MET were administered, the abundance of these gut microbiota recovered to normal levels similar to that of the control group. These results suggested that the increase in *B. pseudolc* and *Parabacteroides distasor* in gut microflora might be the causative factors of T2DM. The growth of these microorganisms was inhibited by 80 mg/kg PSE, and this effect was similar to that of 140 mg/kg MET. Furthermore, 80 mg/kg PSE improved the clinical symptoms of the mice with T2DM induced by long-term HFD. However, 10 mg/kg PSE did not exhibit such effects.

### Beta diversity and cluster analysis of the species composition of the fecal samples of mice in the normal, diabetes, MET, and PSE treatment groups

**Figure 5:**
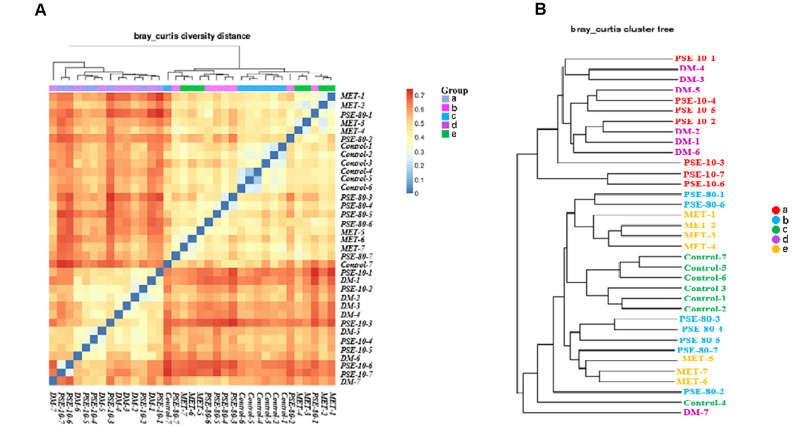
**Effect of PSE on gut microflora of type 2 diabetic mice-induced by high-fat diet.** The Bray Curtis heatmap of beta diversity analysis (**A**) and cluster analysis results of species composition (**B**). Letters a, b, c, d, and e in the graph represent the 10 mg/kg PSE-treated, 80 mg/kg PSE-treated, normal control, diabetes mellitus, and 140 mg/kg MET-treated groups, respectively.

The Bray Curtis heatmap of the results of the beta diversity and cluster analysis of the species composition is shown in Figures 5A and 5B. The similarity of microbial flora among the control group, 80 mg/kg PSE treatment group, and MET treatment group was very high. The gut microflora in the DM and 10 mg/kg PSE-treated groups were similar. Cluster analysis revealed that the microbiota of 80 mg/kg PSE-treated, control, and 140 mg/kg MET-treated groups significantly differed from those of 10 mg/kg PSE-treated and DM groups. These results implied that the imbalance of gut microbiota was associated with T2DM. Therefore, 80 mg/kg PSE and 140 mg/kg MET might significantly affect intestinal microorganisms. However, 10 mg/kg PSE was inefficient.

### LDA effect size analysis of the 16S RNA sequence of intestinal microflora after oral PSE administration

**Figure 6:**
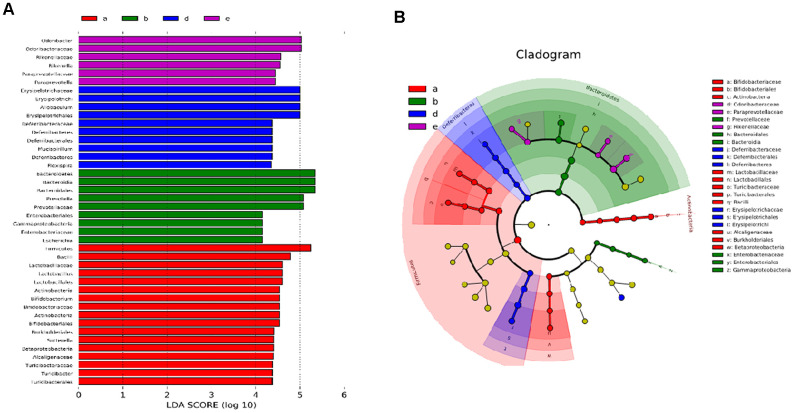
**Effect of PSE on gut microflora of type 2 diabetic mice-induced by high-fat diet.** The LDA score (**A**) and profiling of LEFSE (**B**) analysis. Letters a, b, d, and e in the graph represent 10 mg/kg PSE-treated, 80 mg/kg PSE-treated, the diabetes model, and 140 mg/kg MET-treated groups, respectively.

The linear discriminant analysis (LDA) score and profiling of LDA effect size (LEFSE) analysis are displayed in Figures 6A and 6B. *Erysipelotrichi*, *Allobaculum*, *Erysipelotrichaceae*, *Erysipelotrichales*, *Deferribacteraceae*, *Deferribacterales*, *Deferribacteres*, *Mucispirillum*, and *Flexispira* were the main microorganisms in the DM group. The abundance of *Firmicutes*, *Bacilli*, *Lactobacillaceae*, *Lactobacillales*, *Actinobacteria*, *Bifidobacteriaceae*, *Bifidobacterium*, *Bifidobacterialeacs*, *Sutterella*, *Turicibacteraceae*, *Turicibacter*, *Turicibacterales*, *Alcaligenaceae*, *Burkholderiales*, *Betaproteobacteria*, and *Bataproteo-bacteria* of the 10 mg/kg PSE-treated group significantly increased compared with that of the control group. Conversely, *Bacteroidetes*, *Bacteroidia*, *Bacteroidales*, *Prevotella*, *Prevotellaceae*, *Enterobacteriales*, *Gamma-proteobacteria*, *Enterobacteriaceae*, and *Escherichia* in the 80 mg/kg PSE-treated group were few. By contrast, the *Rikenellaceae*, *Rikenella*, *Odoribacteraceae*, *Paraprevotellaceae*, and *Paraprevotella* in the 140 mg/kg MET-treated group were abundant, and they occupied a high proportion among all the microorganisms. These results suggested that 80 mg/kg PSE and 140 mg/kg MET might regulate the gut microbiota of the mice with T2DM and elicit an antidiabetic effect on mice with T2DM depending on the type of microorganisms.

### PICRUST function analysis of the gut microbiota of mice with T2DM after PSE treatment

**Figure 7:**
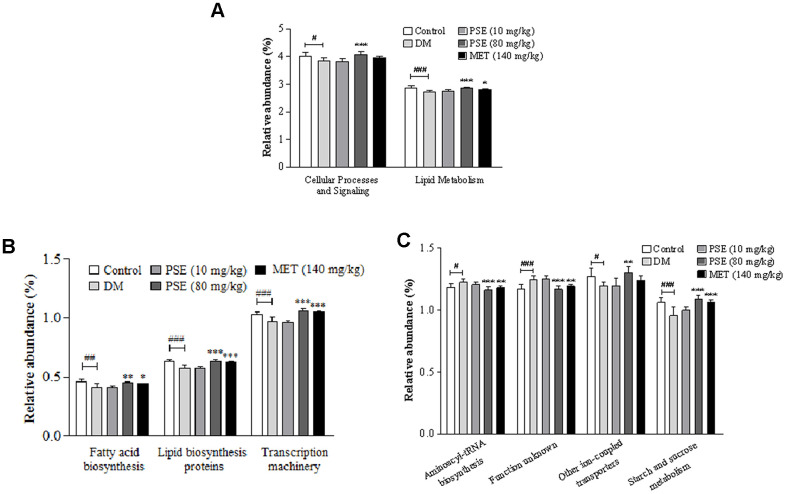
**Results of PICRUST function analysis for gut microbiota of type 2 diabetic mice after administering PSE.** The relative abundance changes of KEGG pathways at level 2 (**A**) and level 3 (**B**, **C**) with PICRUST analysis. Each value was expressed as the mean ± SEM of 7 mice. ^#^, ^##^, and ^###^ represent significant difference compared to the normal control group at p < 0.05, p < 0.01, and p < 0.001, respectively. ^*^, ^**^, and ^***^ indicate significant difference compared to the DM group at p < 0.05, p < 0.01, and p < 0.001, respectively.

PICRUST was applied to determine the 16S RNA sequence and predict the abundance of gut microbiota at levels 2 and 3 of the KEGG pathway. The results of PICRUST function analysis of the gut microbiota of the mice with T2DM are shown in Figure 7, Supplementary Figures 7 and 8. The abundance of the gut microbiota in cellular process signaling and lipid metabolism at level 2 of the KEGG pathway of the DM group significantly decreased compared with that of the normal control group (Figure 7A; p < 0.05 and p < 0.01, respectively). After the treatment with 80 mg/kg PSE, the abundance of cellular process signaling and lipid metabolism of the diabetic model mice significantly increased compared with that of the DM group (Figure 7A; both with p < 0.001). Furthermore, the abundance of the gut microbiota in fatty acid biosynthesis, lipid biosynthesis, proteins, transcription machinery, other ion-coupled transporters, and starch and sucrose metabolism at level 3 of the KEGG pathway in the DM group significantly decreased (Figures 7B and 7C; p < 0.001, p < 0.01, p < 0.01, p < 0.05, and p < 0.001, respectively). The abundance of the gut microflora in aminoacyl-tRNA biosynthesis and an unknown function at the same level in the DM group increased (Figures 7B and 7C; p < 0.05 and p < 0.001, respectively). All these parameters or factors in the mice with T2DM recovered or mostly recovered to normal levels after they were treated with 80 mg/kg PSE and MET at the indicated dose. These results suggested that the gut microorganisms involved in lipid, sugar, and protein metabolism were affected by PSE and MET treatments that caused antidiabetic effects.

### PSE-induced reduction of LPS and pro-inflammatory cytokines in the blood of mice with T2DM

**Figure 8:**
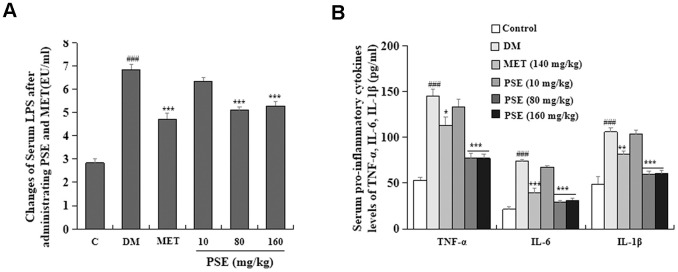
**Effects of PSE on serum endotoxin and pro-inflammatory cytokines of HFD-induced diabetic mice.** The change on serum endotoxin (**A**) and pro-inflammatory cytokines (**B**) of HFD-induced diabetic mice after administering PSE at 10, 80, and 160 mg/kg for 6 weeks, respectively. Each value was expressed as the mean ± SEM of 10 mice. ^###^ represents significant difference compared to the control group at p < 0.001. ^*^, ^**^, and ^***^ indicate significant difference compared to the DM group at p < 0.05, p < 0.01, and p < 0.001, respectively.

Inflammation is one of the important factors that induce T2DM [7–9]. As such, this factor was investigated in our study. The changes in LPS and pro-inflammatory cytokines in the blood of experimental mice are displayed in Figure 8. The LPS (Figure 8A) and pro-inflammatory cytokines (Figure 8B) of DM significantly increased after the long-term feeding of HFD. Simultaneously, the LPS of the mice with T2DM obviously decreased after they were treated with 80 and 160 mg/kg PSE (Figure 8A; both with p < 0.001). However, 10 mg/kg PSE did not significantly affect the LPS in the blood of the mice with T2DM (Figure 8A). Pro-inflammatory cytokines, such as TNF-α, IL-6, and IL-1α, did not significantly change in the mice treated with 10 mg/kg PSE. However, they significantly decreased after the treatment with 80 mg/kg and 160 mg/kg PSE and with the indicated dose of MET (p < 0.001, p < 0.001, p < 0.001, p < 0.05, p < 0.001, and p < 0.01, respectively). These results indicated that PSE and MET could significantly prevent the increase in LPS and pro-inflammatory cytokines in the blood of the mice with T2DM induced by HFD. However, low-dose PSE (10 mg/kg) was not effective against these factors.

### PSE-induced reduction of inflammation-related gene expression and protein levels in the adipose tissues and liver of mice with T2DM

**Figure 9:**
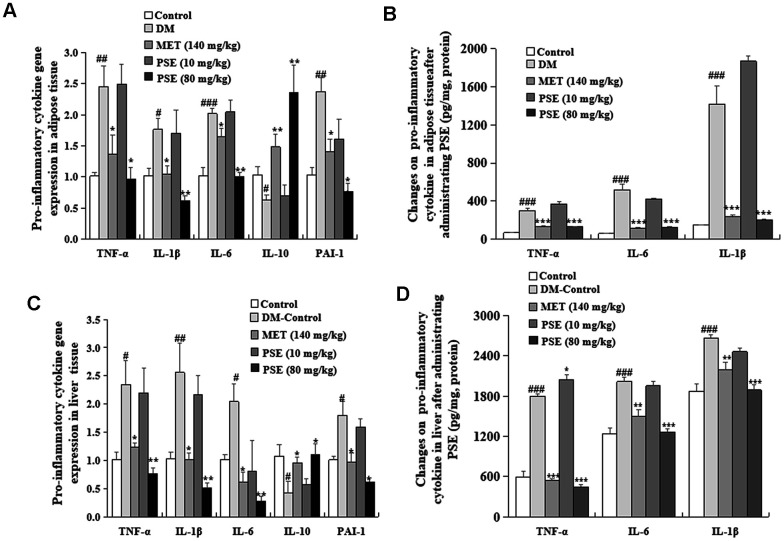
**Effects of PSE on pro-inflammatory cytokine gene expression and pro-inflammatory cytokine protein level in the liver and adipose tissue of HFD-induced diabetic mice.** The change in pro-inflammatory cytokine gene expression (**A**) and pro-inflammatory cytokine at the protein level (**B**) in the liver of HFD-induced diabetic mice after administering PSE at 10, 80, and 160 mg/kg for 6 weeks, respectively. The change in pro-inflammatory cytokine gene expression (**C**) and pro-inflammatory cytokine at the protein level (**D**) in the adipose tissue of HFD-induced diabetic mice after administering PSE at 10, 80, and 160 mg/kg for 6 weeks, respectively. Each value was expressed as the mean ± SEM of 10 mice. ^#^, ^##^, and ^###^ represent significant difference compared to the control group at p < 0.05, p < 0.01, and p < 0.001, respectively. ^*^, ^**^, and ^***^ indicate significant difference compared to the DM group at p < 0.05, p < 0.01, and p < 0.001, respectively.

The changes in inflammation-related gene expression and protein levels in adipose tissues are displayed in Figures 9A and 9B. The gene expression levels of TNF-α, IL-1β, IL-6, and PAI-1 in the adipose tissues of the DM group were higher than those of the normal control mice (Figure 9A; p < 0.05, p < 0.01, p < 0.001, and p < 0.01, respectively). These gene expression levels in the mice with T2DM decreased after they were treated with 80 mg/kg PSE and 140 mg/kg MET (Figure 9A; p < 0.05, p < 0.01, p < 0.001, p < 0.05, and p < 0.001, respectively). Furthermore, the protein levels of TNF-α, IL-6, and IL-1β were examined (Figure 9B). These results strongly supported the conclusion from the mRNA analysis. The effect of PSE on inflammation in the liver was also investigated. The changes in inflammation-related gene expression and protein levels are shown in Figures 9C and 9D. The significant increase and decrease in the mRNA expression and protein levels of these factors in the liver were similar to those in the adipose tissue (p < 0.05, p < 0.01, and p < 0.001). Interestingly, the IL-10 gene expression in the tissues in the DM group increased after the mice were treated with 80 mg/kg PSE and 140 mg/kg MET (Figures 9A and 9C; p < 0.01 and p < 0.001, respectively). These results indicated that PSE alleviated inflammation in the adipose tissue and liver of mice with T2DM induced by HFD.

### PSE-induced alleviation of the inflammation of the hypothalamus of mice with T2DM

**Figure 10:**
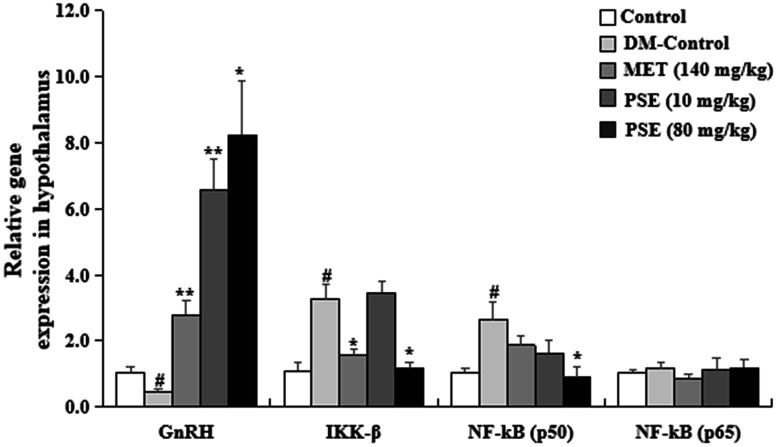
**Effects of PSE on relative gene expression in the hypothalamus of HFD-induced diabetic mice.** The change on relative gene expression in the hypothalamus of HFD-induced diabetic mice after administering PSE at 10, 80, and 160 mg/kg for 6 weeks. Each value was expressed as the mean ± SEM of 10 mice. ^#^ represents significant difference compared to the control group at p < 0.05. ^*^ and ^**^ indicate significant difference compared to the DM group at p < 0.05 and p < 0.01, respectively.

Hypothalamic immunity is mediated by IkappaB kinase-β (IKK-β) and nuclear factor kappaB (NF-κB). Hypothalamus combatant aging-related health problem is restored via immune-neuroendocrine integration and immune inhibition or gonadotropin-releasing hormones (GnRH) [26]. Thus, changes in GnRH, IKK-β, and NF–Kβ gene expression in the hypothalamus were examined in our study. The results are displayed in Figure 10. The mRNA abundance of IKK-β and NF-Kβ (p50) in the hypothalamus of the mice with T2DM was higher than that of the normal control mice (p < 0.05 and p < 0.05, respectively). The gene expression level of GnRH was lower than that of the normal mice (p < 0.05). No significant difference in the NF-Kβ (p65) gene expression between the normal and diabetic mice was observed. Importantly, the gene expression levels of IKK-β and NF-Kβ (p50) in the hypothalamus of the mice with T2DM significantly decreased after they were treated with 80 mg/kg PSE (p < 0.05 and p < 0.05) and 140 mg/kg MET (p < 0.05). Interestingly, the expression levels of GnRH in the hypothalamus of the mice with T2DM obviously increased after the treatment with 10 and 80 mg/kg PSE and 140 mg/kg MET (p < 0.01, p < 0.01, and p < 0.01, respectively). These results revealed that PSE prevented the inflammation of the hypothalamus by regulating the IKK-β/NF-Kβ/GnRH signaling pathway.

## DISCUSSION

In our previous studies, the mixture of PSE and fish oil improves the memory of mice, and PSE has an anti-obesity effect on mice with obesity [15, 22]. The composition analysis of PSE [15] has shown that the PSE obtained in our study is enriched with polyphenols and polysaccharides. Many polyphenols and polysaccharides derived from plants exhibit antidiabetic effects [23–24]. The regulation of intestinal microflora and anti-inflammation is closely related to diabetes [1, 7]. As such, the effect of PSE on mice with T2DM was investigated, and the mechanism of action of PSE as an antidiabetic was clarified by examining the variation of gut microbiota and inflammatory factors.

In the present study, mice with dietary-induced T2DM were used to examine the antidiabetic effect of PSE and its mechanism of action. Figures 1–10 show that PSE improved the clinical symptoms of the mice with T2DM by regulating the composition and ratio of intestinal microbiota and by reducing inflammatory responses. In particular, 80 mg/kg PSE significantly regulated the gut microflora of the mice with T2DM, but a lower dose of 10 mg/kg PSE did not achieve the same result. Importantly, the pharmacodynamic results of PSE were good and consistent with those of gut microbiota analysis in this study.

As reported, feeding animals with HFDs for a long time can induce the dysbiosis of the gut microbiota and increase the levels of LPS in the blood [27]. A large amount of LPS produced by Gram-negative bacteria increases in the digestive tract of mice-fed HFD [28–29]. In a state of intestinal dysbiosis, the integrity of the intestinal membrane is damaged, and the LPS of Gram-negative bacteria enters blood circulation. LPS in the blood may induce systemic inflammation in the peripheral tissues of HFD-fed mice by activating TLR4 signaling in these tissues [30]. Therefore, two key aspects, namely, gut microbiota and inflammatory responses, were considered in the analysis of the mechanism of action. Figures 3–10 and Supplementary Figure 2 reveal that PSE improved the integrity of the gut barrier, alleviated endotoxemia, and decreased the inflammation in the mice with T2DM induced by HFD. The beneficial effects of PSE treatment might be attributed to specific changes in the gut microbiota and the maintenance of intestinal integrity.

The gut microbiota of mice with T2DM is associated with a decrease in the abundance of intestinal *Bacteroides* and an increase in the abundance of *Firmicutes*, indicating that these phyla may participate in diabetes-related inflammation [31]. In our study, the ratio of *Bacteroides* to *Firmicutes* in the mice with HFD-induced T2DM was significantly restored to the normal level after they were treated with 80 mg/kg PSE and 140 mg/kg MET (Figure 4B). The increased abundance of *Actinobacteria* in the DM group decreased in the groups treated with 80 mg/kg PSE and 140 mg/kg MET. This evidence suggested that PSE exhibited antidiabetic effects by altering the ratio of *Bacteroides* to *Firmicutes* and regulating the levels of other intestinal flora. The 16S RNA sequencing analysis of the fecal microflora after the mice were treated with PSE revealed that the potentially harmful gut microorganisms were *Clostridium*, *Actinobacteria*, and *Proteobacteria*. *Bifidobacterium*, *Eubacterium*, and *Fusobacterium*, which produce short-chain fatty acids, are beneficial to health.

The central nervous system controls the metabolism of the whole body by sensing and responding to the circulating signals of nutrient and hormone levels. Clarifying the neuronal mechanisms by which the central nervous system regulates metabolism is a fundamental step in the treatment of metabolic diseases. The hypothalamus is an important region that adjusts metabolism. The ventral medial nucleus of the hypothalamus controls glucose and energy balance. As such, inflammation associated with the hypothalamus was analyzed in this study. Chronic-grade inflammation is involved in the development of T2DM [31–32]. LPS derived from pathogenic Gram-negative bacteria has a critical role in chronic inflammation related to T2DM. LPS activates the toll-like receptor 4 (TLR4) signaling pathway to trigger pro-inflammatory cytokines, such as IL-1, IL-6, and TNF-α, which are the risk factors causing the development of T2DM [33–34]. Therefore, our study also focused on inflammatory responses to investigate the mechanism of action of PSE. The following observations indicated that PSE exhibited antidiabetic effects by inhibiting inflammation in the peripheral tissues and the central nervous system: changes in the LPS and pro-inflammatory cytokines in the plasma (Figure 8), changes in the pro-inflammatory cytokines at the gene expression and protein levels in the liver (Figures 9A and 9B) and adipose tissues (Figures 9C and 9D), and changes in IKK-β and NF-Kβ in the hypothalamus (Figure 10) of the mice with T2DM after the treatment with PSE. The results obtained in this study were consistent with those of other studies [34].

The abundance of Cyanobacteria is negatively correlated with IL-6, TNF-α, and IL-17A but positively correlated with IL-10 [35]. The abundance of *Deferribacteres* and *Tenericutes* at the phylum level is positively correlated with IL-6 and TNF-α; similarly, the abundance of *Mucispirillum* and *Ruminococcaceae*-6 at the genus level is positively correlated with IL-17A and LPS [32]. In the present study, the significant reduction of the abundance of *Cyanobacteria* in the DM group was notably reversed after the treatment with 80 mg/kg PSE (Figures 4A and 4B). The significantly increased abundance of *Mucispirillum* at the genus level in the T2DM group was decreased by 80 mg/kg PSE (Supplementary Figure 5). Moreover, the improvement of IL-6, TNF-α genes expression, and LPS level in the liver and adipose tissue and IKK-β, NF-Kβ, and GnRΗ genes expression in the hypothalamus was examined. However, a significant change in the abundance of Ruminococcaceae-6 was not observed. These results further supported the idea that inflammation in T2DM was closely correlated with the composition of gut microbiota. Thus, PSE elicited an antidiabetic effect by altering the gut microbiota and inhibiting inflammation.

Interestingly, although PSE and MET exhibited antidiabetic effects on the mice with T2DM, the gut microbiota composition of the mice in the PSE- and MET-treated groups remarkably differed. PSE primarily affected the abundance of *Rikenellaceae*, *Odoribacteraceae*, *Paraprevotellaceae*, *Prevotellaceae*, *Bacteroidales*, and *Bacteroidia* in the gut microflora to induce an antidiabetic effect. *Erysipelotrichaceae, Erysipelotrichales, and Erysipelotrichi* in the 140 mg/kg MET-treated group had important roles (Figure 5). These results suggested that different treatments might cause different regulatory effects on the gut microbiota but might eventually achieve similar treatment effects. However, these mechanisms should be further elucidated. In our study, PSE could improve the symptoms of mice with T2DM by maintaining the homeostasis of gut microbiota. However, no direct evidence was obtained to verify this finding. Intensive studies will be conducted in the near future by using antibiotics to treat HFD-induced obesity in mice before oral PSE administration to obtain relevant data.

The possible side effects of long-term administration should be examined to evaluate the potential of PSE. Thus, the level of biochemical markers on toxicity were measured, and the changes in plasma ALT and AST levels (Supplementary Table 3) indicated that PSE was safe for mice.

In conclusion, a HFD-induced T2DM mouse model was utilized to investigate the antidiabetic effect of PSE and its mechanism of action. PSE ameliorated the symptoms of mice with T2DM by regulating the gut microbiota and inhibiting inflammation in the peripheral tissues and central nervous system. Peanuts are common food worldwide, and PSE used in this study was obtained from peanut skin, which is enriched with polyphenol and polysaccharides. PSE elicited the same effect as MET, which is the first-line drug for diabetes, on mice with HFD-induced T2DM. Therefore, PSE could be developed as a drug or an adjuvant to prevent or treat T2DM.

## MATERIALS AND METHODS

### Preparation of PSE

Dried peanut skin (500 g; Anji County, Huzhou City, Zhejiang Province, China) was powdered and then extracted with a mixture of ethanol and water at a ratio of 40:60 three times. The extracts were collected, and ethanol was evaporated to give a water solution applied to HP-20 resin. The sample was washed with water and eluted with 40% aqueous ethanol. The obtained fractions were combined, concentrated, and dried by freeze-drying to give a total of 57.5 g of PSE powder (11.5% of dry weight). PSE components were analyzed through high-performance liquid chromatography [15]. The stability of PSE was also examined by comparing the properties of the ingredients before and after the experiment. When the animal tests were completed, the remaining PSE was evaluated through ^1^H NMR and HR-ESI-MS. No changes were detected.

### Construction of T2DM mouse model and animal experimental design

Five-week-old male mice from Institute for Cancer Research were purchased from the Zhejiang Academy of Medical Sciences (Hangzhou, China). They were divided into five mice per cage and kept under controlled conditions: temperature of 22 ± 1 °C, humidity of 50% ± 5%, and under 12 h light/dark cycle. They were fed with a commercial diet (normal diet [ND] or HFD with 22.3% crude protein, 19.8% fat, and 44.6% carbohydrate from SLAC Experimental Animal Co., Ltd., Shanghai, China) for 4 months and allowed to take food and water *ad libitum*. During the animal experiments, water consumption, food intake, and body weight were monitored weekly. After the mice were fed with HFD for 4 months, their fasting blood glucose level was measured. The T2DM mouse model was considered to be successfully established when the following symptoms were observed or measured simultaneously: >7 mmol/L blood glucose concentration, obesity, polydipsia, polyphagia, and polyuria.

Subsequently, 10 normal mice and 50 mice with diet-induced diabetes were randomly divided into six groups: normal mouse group (control), DM control group, diabetic model with MET treatment as a positive control, and DM with PSE treatment. The sample was dissolved in water and administered orally. The control group was fed with ND, whereas the other groups were given HFD during experiments. The control and DM groups were given water as a vehicle every day. The positive control group received MET (Aladdin Bio-chem Technology Company, Shanghai, China) at a dose of 140 mg/kg per day. The PSE-treated groups were given doses of 10, 80, and 160 mg/kg per day. MET and PSE were administered daily for 6 weeks. Fasting blood glucose, body weight, food intake, and water consumption were measured at the end of each week. At the end of the experiment, the mice were starved overnight and sacrificed via neck dislocation under deep anesthesia by using ether. The blood, heart, liver, pancreas, spleen, kidney, adipose tissue, and hypothalamus were removed, weighed, and stored at −30 °C for future analysis. This experiment was repeated twice and performed in accordance with international ethical standards with guidance and approval from the Committee of Experimental Animal Care, Zhejiang University (Permit Number: ZJU20160436).

### Enzyme-linked immunosorbent analysis

Blood samples were collected from the orbital sinus. The samples were allowed to stand at room temperature for 2 h, centrifuged at 12,000 × *g* for 15 min at 4 °C to obtain the plasma, and stored at −30 °C until use for biochemical analysis. TNF-α, IL-1β, and IL-6 protein levels in the plasma, liver, and adipose tissue were measured using commercially available mouse TNF-α, IL-1β, and IL-6 ELISA kits (4A Bio-Tech, Beijing, China) in accordance with the manufacturer’s instructions.

### Detection of fasting blood glucose and plasma biochemical indicators at the end of the experiment

At the end of each week, the mice were fasted, and the fasting blood glucose level was measured from the blood collected from the tip of the tail vein by using a glucometer (Andon Health, Tianjin, China). The plasma samples at the end of the experiment were sent to Hangzhou Dian Medical Test Center (Hangzhou City, Zhejiang Province, China) to evaluate the levels of alanine aminotransferase (ALT), aspartate aminotransferase (AST), triglycerides (TGs), total cholesterol (TC), and glucose in the plasma with a biochemical analyzer (COBAS 8000; Roche, Basel, Switzerland) and assay kits (Roche, Basel, Switzerland).

### Oral glucose and insulin tolerance tests

In this experiment, glucose tolerance was measured after PSE was administered for 5 weeks. Overnight-fasted mice were given glucose orally at a dose of 1.5 g/kg. The glucose concentrations in the blood taken from the tail vein at 0, 15, 30, 60, 90, and 120 min were measured with a glucometer.

Subsequently, an ITT was performed. The mice were fasted for 4 h. Afterward, insulin (Yeason, Shanghai, China) was intraperitoneally administered at a dose of 0.4 units/kg. Then, glucose concentration was determined using the blood samples from the tail vein at 0, 15, 30, 45, and 60 min.

### Analysis through real-time polymerase chain reaction

The liver and epididymal fat were removed surgically, weighed, quickly frozen, and kept at −30 °C until RNA extraction. RNA from the white adipose tissue, liver, and hypothalamus was extracted, and their cDNA was synthesized [36]. Approximately 100 mg of epididymal fat, 50 mg of liver tissue, and one hypothalamus were used for RNA extraction with a Trizol reagent (Invitrogen, California, USA). Afterward, 2.5 μg of the total RNA of each sample was used for reverse transcription by using a HiFi-MMLV cDNA kit (Fermentas, Shenzhen, China). Real-time PCR analysis was performed using CFX96-Touch (Bio-rad, Hercules, CA, USA) and SYBR Premix EX Taq^TM^ (Takara, Otsu, Japan). The primer sequences (Sangon Biotech Co., Ltd., Shanghai, China) used in PCR are given in Supplementary Table 1. Glyceraldehyde-3-phosphate dehydrogenase was used as an internal control for normalization.

### Analysis of gut microbiota

The feces of the mice in each group was taken at the end of the animal experiments to detect the change in gut microflora. Stool samples were quickly frozen in liquid nitrogen and sent to BGI Company (Wuhan, China) for the analysis of gut microbiota in accordance with a previously described method [1]. The analysis method is also presented in Supplementary Information.

### Statistical analysis

Animal experiments were repeated twice. Data were expressed as the mean ± standard error of the mean and statistically analyzed through two-tailed multiple t tests with GraphPad Prism 5 and a Kruskal test or a Wilcoxon test with Metastats. Data about gut microflora were examined using the R software. Results with p < 0.05 were considered significantly different between groups.

### Statement of ethics

This experiment was repeated twice and performed according to international ethical standards with guidance and approval from the Committee of Experimental Animal Care, Zhejiang University (Permit Number: ZJU20160436).

## Supplementary Material

Materials and Methods

Supplementary Figures

Supplementary Tables
