# Supplementary material for: Peanut skin extract ameliorates the symptoms of type 2 diabetes mellitus in mice by alleviating inflammation and maintaining gut microbiota homeostasis
Source: Aging (Albany NY). 2020 Jul 22;12(14):13991–4018. doi: 10.18632/aging.103521 (PMC7425515; doi:10.18632/aging.103521)
Supplement: Materials and Methods [file aging-12-103521-s001..pdf]

SUPPLEMENTARY MATERIALS

MATERIALS AND METHODS

Gut microbiota analysis

Briefly, DNA was extracted using MagPure Stool DNA Kit (Magen Bioscience Company, Shanghai, China). For each sample, the 16S rRNA gene V4 regions were amplified using a composite sense primer and anti-sense primer containing a unique 10-base barcode to tag each PCR product. The PCR reaction conditions are as follows: 95 °C for 3 min, followed by 15-25 cycles of 98 °C for 20 s, 45 °C for 15 s and 72 °C for 15 s, and a final extension of 72 °C for 1 min. The composite primer pairs consisted of the sense primer (515F: GTGCCAGCMGCCGCGGTAA), and the anti-sense primer (806R: GGACTACHVGGGTWTCTAAT). The target Amplicon fragment was recovered, and the broken sticky end was repaired to a flat end by T4 DNA Polymerase, Klenow DNA Polymerase and T4 PNK, and then a base “A” was added at the 3’end to enable the DNA fragment to carry a “T” base with the 3’ end. Special junction connection; or design and synthesize double Index fusion primers containing sequencing junctions, using genomic DNA as template for fusion primer PCR, Replicate PCRs were pooled and amplicons were purified using magnetic beads. High quality reads for bioinformatics analysis were selected and all of the effective reads from all samples were clustered into OTUs based on 97% sequence similarity according to UPARSE. Alpha diversity analysis such as rarefaction analysis and Shannon index were taken using R software (v3.1.1). Beta diversity analysis and Fast UniFrac PCoA were performed with the phylogenetic tree constructed by inserting the representative of each OTU generated by using QIIME (v1.80). Meanwhile, LEFSE (LDA Effect Size) analysis and PICRUST (Phylogenetic Investigationof Communities by Reconstruction of Unobserved States) analysis were done with Lefse and PIRCUST softwares, respectively. The difference of microbial community abundance between the two groups was tested by statistical method, and the significant difference was evaluated by FDR (false discovery rate). From the test results, the species that lead to the difference in the composition of the two groups of samples can be screened out. In our analysis, significant differences between groups were analyzed in the phylum, class, order, family, genus and species classification grades.
