# Supplementary material for: Peanut skin extract ameliorates the symptoms of type 2 diabetes mellitus in mice by alleviating inflammation and maintaining gut microbiota homeostasis
Source: Aging (Albany NY). 2020 Jul 22;12(14):13991–4018. doi: 10.18632/aging.103521 (PMC7425515; doi:10.18632/aging.103521)
Supplement: Supplementary Figures [file aging-12-103521-s003..pdf]

SUPPLEMENTARY FIGURES

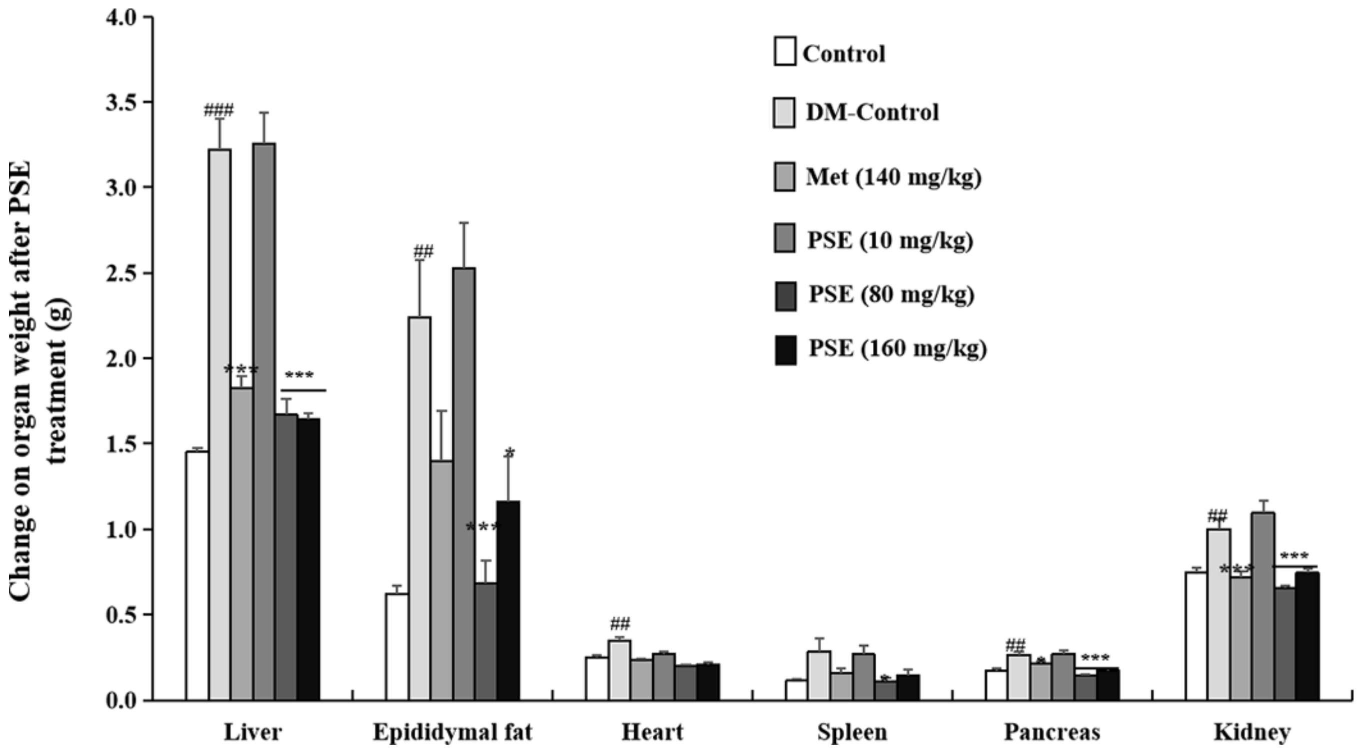

Supplementary Figure 1. Effect of PSE on the organs weight of type 2 diabetic mice. Each value was expressed as the mean ± SEM of 10 mice. ## and ### represent significant difference comparison with the control group at p< 0.01 and p< 0.001. * and *** indicate significant difference comparison with the DM-C group at p< 0.05, and p< 0.001.

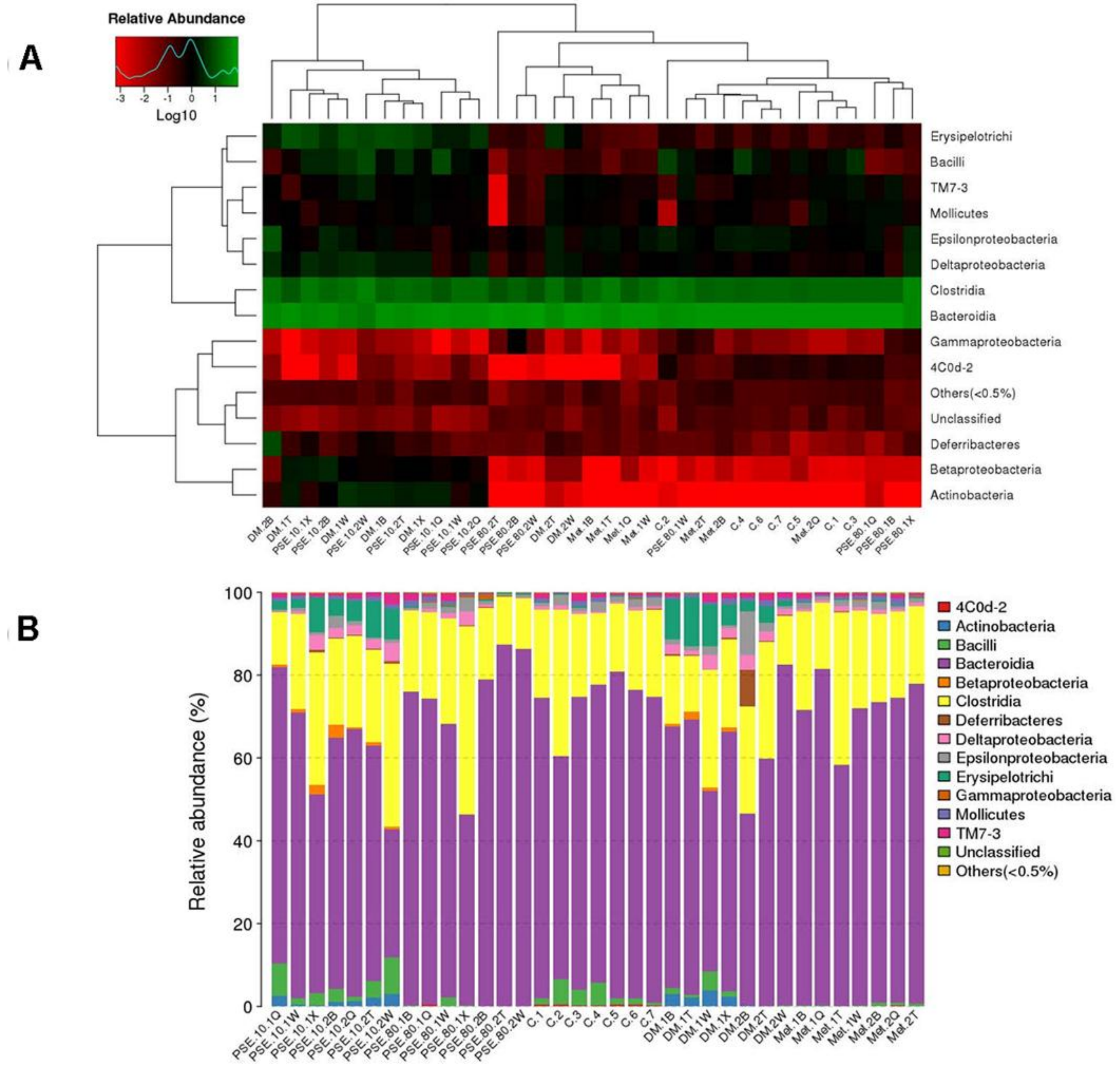

Supplementary Figure 2. Effect of PSE on gut microflora of type 2 diabetic mice induced by high-fat diet. Heat map of classes level species abundance (**A**). Histogram of species profiling at class classification level (**B**). Column height represents relative abundance.

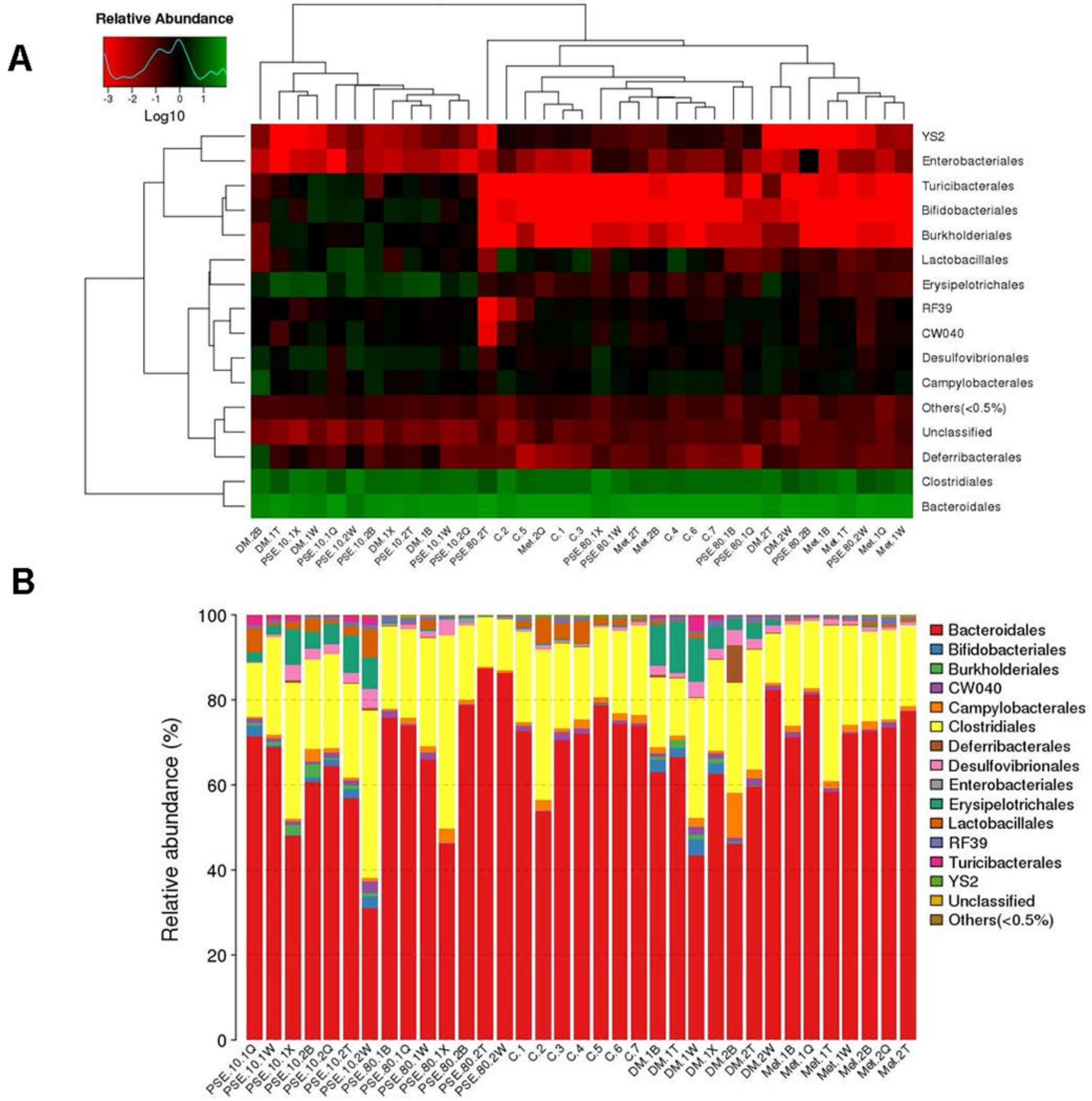

Supplementary Figure 3. Effect of PSE on gut microflora at order level of type 2 diabetic mice induced by high-fat diet. Heat map of order level species abundance (**A**). Histogram of species profiling at order classification level (**B**). Column height represents relative abundance.

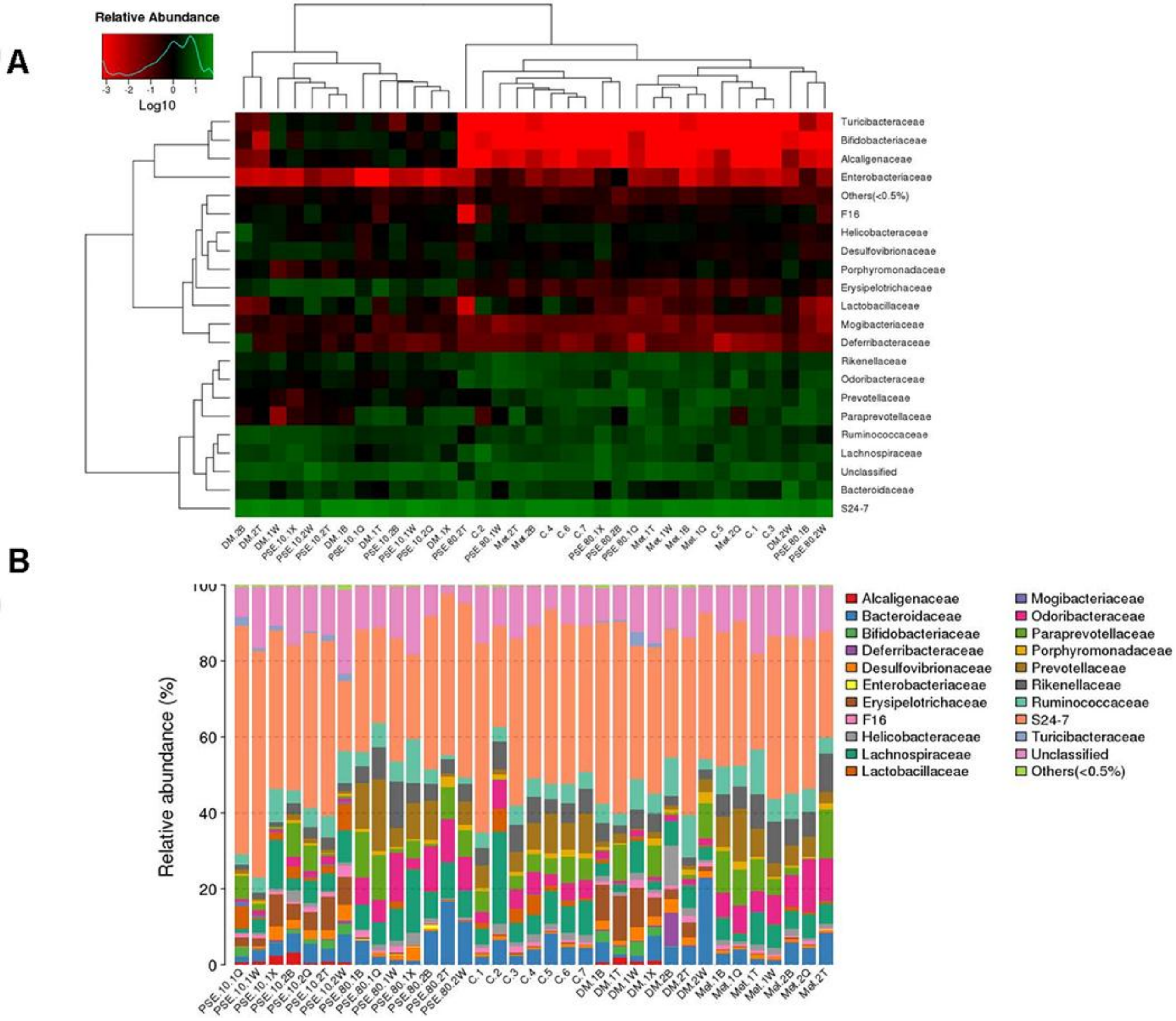

Supplementary Figure 4. Effect of PSE on gut microflora at family level of type 2 diabetic mice induced by high-fat diet. Heat map of family level species abundance (**A**). Histogram of species profiling at order classification level (**B**). Column height represents relative abundance.

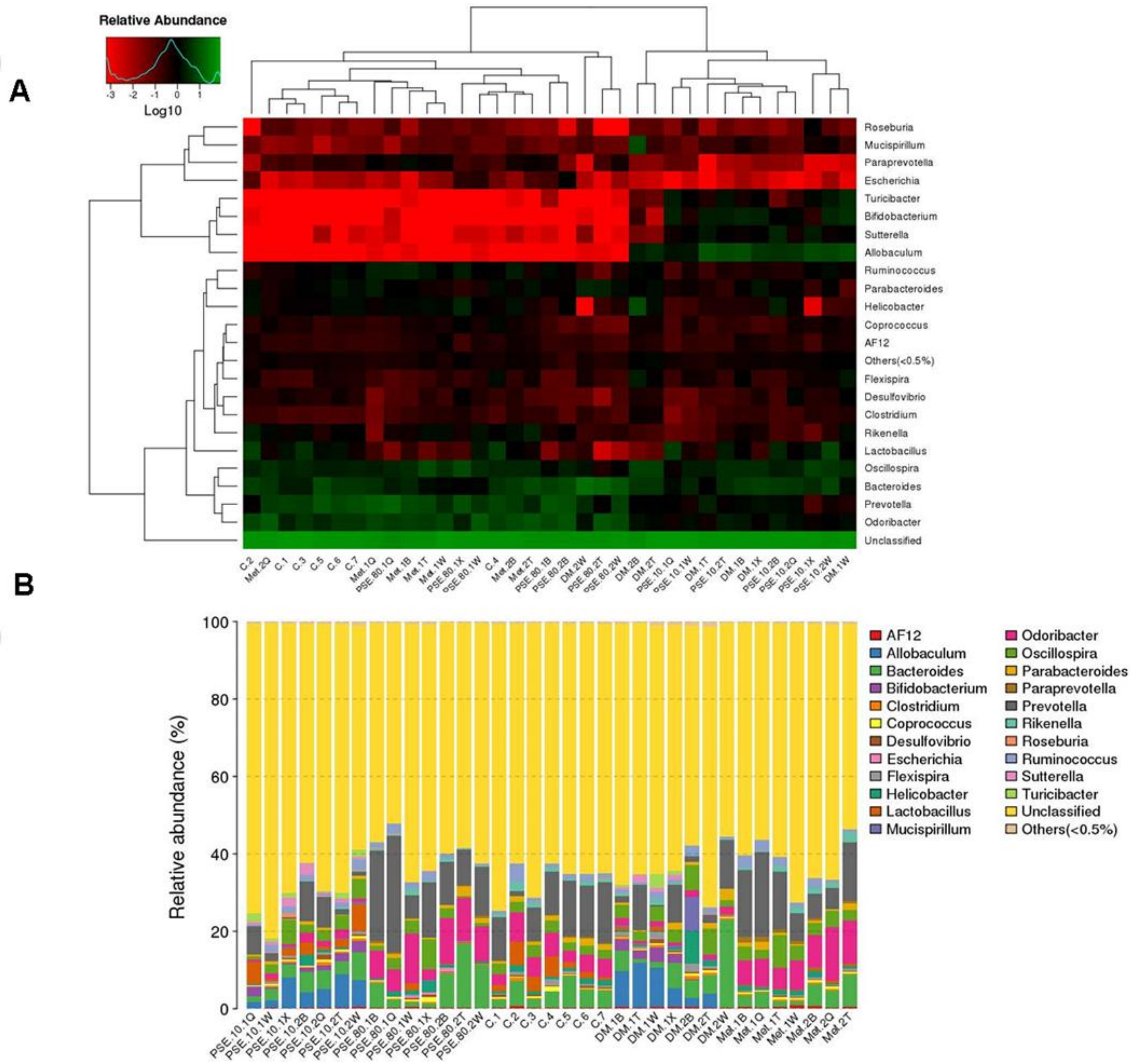

Supplementary Figure 5. Effect of PSE on gut microflora at genus level of type 2 diabetic mice induced by high-fat diet. Heat map of genus level species abundance (**A**). Histogram of species profiling at genus classification level (**B**). Column height represents relative abundance.

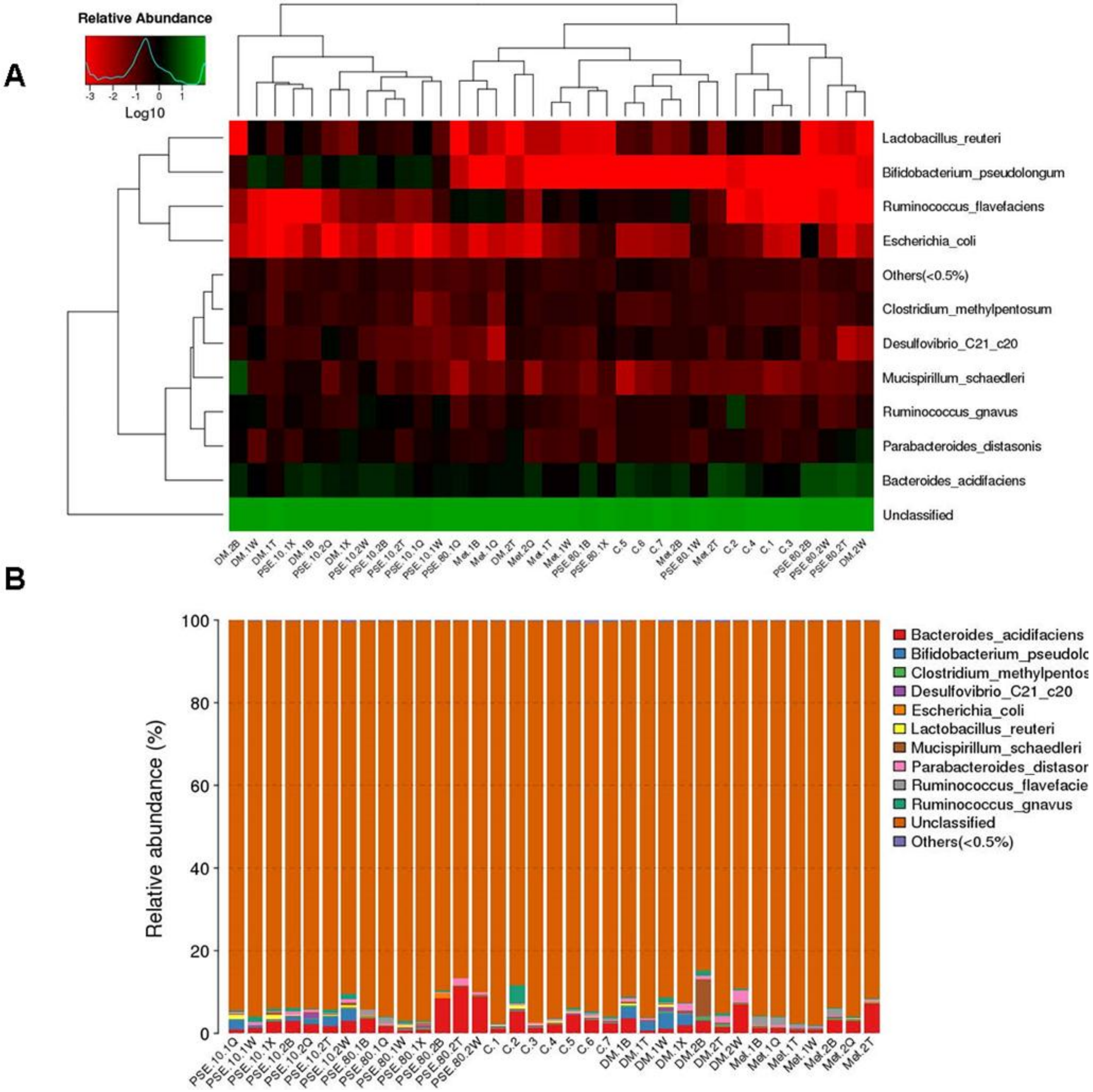

Supplementary Figure 6. Effect of PSE on gut microflora at species level of type 2 diabetic mice induced by high-fat diet. Heat map of order level species abundance (**A**). Histogram of species profiling at order classification level (**B**). Column height represents relative abundance.

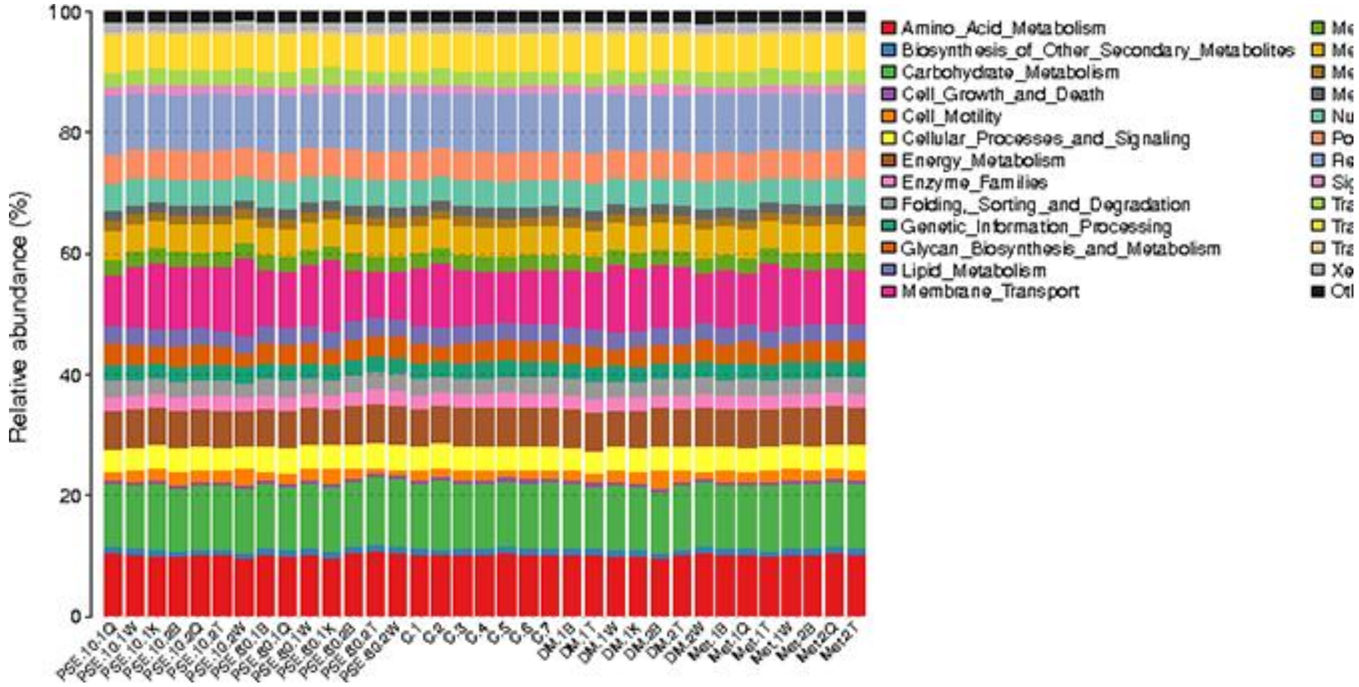

Supplementary Figure 7. Histogram of species profiling for KEGG pathways at level 2 with PICRUST analysis. The same colour represented same primary pathway. Column height represents relative abundance.

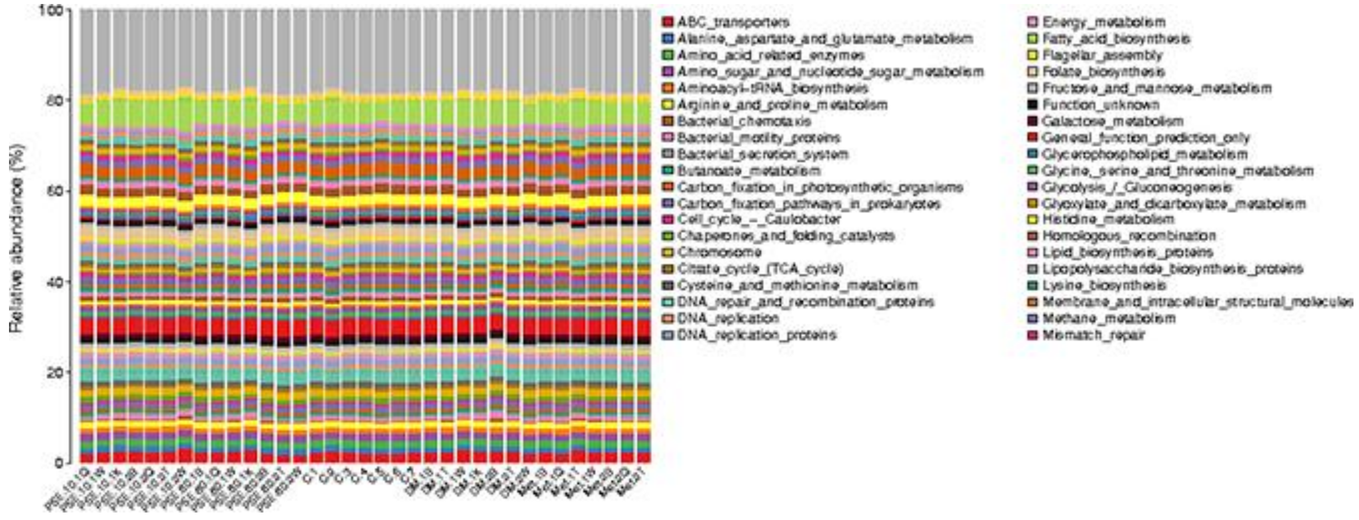

Supplementary Figure 8. Histogram of species profiling for KEGG pathways at level 3 with PICRUST analysis. The same colour represented same primary pathway. Column height represents relative abundance.
