# Supplementary material for: Peanut skin extract ameliorates the symptoms of type 2 diabetes mellitus in mice by alleviating inflammation and maintaining gut microbiota homeostasis
Source: Aging (Albany NY). 2020 Jul 22;12(14):13991–4018. doi: 10.18632/aging.103521 (PMC7425515; doi:10.18632/aging.103521)
Supplement: Supplementary Tables [file aging-12-103521-s002..pdf]

SUPPLEMENTARY TABLES

Supplementary Table 1. Primers sequence in RT-PCR analysis of this study.

Gene	Species	Sequences
TNF-α	mouse	sense: 5'-TAGCCAGGAGGGAGAACAGA-3'
	mouse	anti-sense: 5'-TTTTCTGGAGGGAGATGTGG-3'
IL-6	mouse	sense: 5'-CCGGAGAGGAGACTTCAC-3'
	mouse	anti-sense: 5'-TCCACGATTTCCCAGAGA-3'
IL-1β	mouse	sense: 5'-TTGAAGAAGAGCCCATCCTC-3'
	mouse	anti-sense: 5'-CAGCTCATATGGGTCCGAC-3'
IL-10	mouse	sense: 5'-GCTCTTACTGACTGGCATGAG-3'
	mouse	anti-sense: 5'-CGCAGCTCTAGGAGCATGTG-3'
PAI-1	mouse	sense: 5'-TCAGCCCTTGCTTGCCTCAT-3'
	mouse	anti-sense: 5'-GCATAGCCAGCACCGAGGA-3'

Supplementary Table 2. Effect of PSE on blood biochemical indicators of type 2diabetic mice.

	ALT(U/L)	AST(U/L)	TG(mmol/L)	TC(mmol/L)
**Control**	31.28 ± 2.18	121.82 ± 11.34	1.41 ± 0.08	3.32 ± 0.22
**DM-Control**	32.04 ± 4.36	110.09 ± 31.55	2.47 ± 0.30##	4.52 ± 0.27#
**MET(140 mg/kg)**	34.41 ± 3.04	99.11 ± 3.93	1.66 ± 0.10*	3.13 ± 0.24**
**PSE (80 mg/kg)**	33.81 ± 2.46	110.90 ± 7.14	1.43 ± 0.15**	3.46 ± 0.30*

The sample number is 7.

ALT, Alanine aminotransferase; AST, Aspartate aminotransferase; TC, Total cholesterol; TG, Triglycerides, PSE, Peanut skin extract; MET, metformin

##, ### and *,** represent significant difference ##and ### represent significant difference compared with the control group at $p < 0.01$, and $p < 0.001$, respectively. * and ** indicate significant difference compared with the DM group at $p < 0.05$, $p < 0.01$, respectively.

Supplementary Table 3. The sample OTU statistics of faeces of diabetic mice after treatment PSE and metformin.

Sample Name	Tag Number	OTU Number
C.1	74670	545
C.2	73564	401
C.3	71312	534
C.4	71872	543
C.5	73304	508
C.6	72836	550
C.7	70953	564
DM.1B	75035	542
DM.1T	75490	485
DM.1W	76799	512
DM.1X	73047	520
DM.2B	73409	538
DM.2T	74625	562
DM.2W	71626	418
MET.1B	68754	546
MET.1Q	72398	459
MET.1T	73173	568
MET.1W	73552	557
MET.2B	71234	522
MET.2Q	71195	502
MET.2T	72345	514
PSE.10.1Q	74698	460
PSE.10.1W	74063	529
PSE.10.1X	72384	483
PSE.10.2B	71702	506
PSE.10.2Q	74663	543
PSE.10.2T	71050	561
PSE.10.2W	70903	564
PSE.80.1B	71278	434
PSE.80.1Q	70671	496
PSE.80.1W	73085	540
PSE.80.1X	73783	530
PSE.80.2B	73167	391
PSE.80.2T	73783	280
PSE.80.2W	69909	397

* The sample numbers of each group are 7; C, DM, MET and PSE represented control group, diabetic group, metformin-treated group, PSE-treated group.
